# Photobleaching of Chlorophyll in Light-Harvesting Complex II Increases in Lipid Environment

**DOI:** 10.3389/fpls.2020.00849

**Published:** 2020-06-24

**Authors:** Mónika Lingvay, Parveen Akhtar, Krisztina Sebők-Nagy, Tibor Páli, Petar H. Lambrev

**Affiliations:** ^1^Institute of Plant Biology, Biological Research Centre, Szeged, Hungary; ^2^Doctoral School of Physics, Faculty of Science and Informatics, University of Szeged, Szeged, Hungary; ^3^Institute of Biophysics, Biological Research Centre, Szeged, Hungary

**Keywords:** electron paramagnetic resonance, non-photochemical quenching, photoinhibition, photosystem II, reconstituted membranes, singlet oxygen

## Abstract

Excess light causes damage to the photosynthetic apparatus of plants and algae primarily via reactive oxygen species. Singlet oxygen can be formed by interaction of chlorophyll (Chl) triplet states, especially in the Photosystem II reaction center, with oxygen. Whether Chls in the light-harvesting antenna complexes play direct role in oxidative photodamage is less clear. In this work, light-induced photobleaching of Chls in the major trimeric light-harvesting complex II (LHCII) is investigated in different molecular environments – protein aggregates, embedded in detergent micelles or in reconstituted membranes (proteoliposomes). The effects of intense light treatment were analyzed by absorption and circular dichroism spectroscopy, steady-state and time-resolved fluorescence and EPR spectroscopy. The rate and quantum yield of photobleaching was estimated from the light-induced Chl absorption changes. Photobleaching occurred mainly in Chl *a* and was accompanied by strong fluorescence quenching of the remaining unbleached Chls. The rate of photobleaching increased by 140% when LHCII was embedded in lipid membranes, compared to detergent-solubilized LHCII. Removing oxygen from the medium or adding antioxidants largely suppressed the bleaching, confirming its oxidative mechanism. Singlet oxygen formation was monitored by EPR spectroscopy using spin traps and spin labels to detect singlet oxygen directly and indirectly, respectively. The quantum yield of Chl *a* photobleaching in membranes and detergent was found to be 3.4 × 10^–5^ and 1.4 × 10^–5^, respectively. These values compare well with the yields of ROS production estimated from spin-trap EPR spectroscopy (around 4 × 10^–5^ and 2 × 10^–5^). A kinetic model is proposed, quantifying the generation of Chl and carotenoid triplet states and singlet oxygen. The high quantum yield of photobleaching, especially in the lipid membrane, suggest that direct photodamage of the antenna occurs with rates relevant to photoinhibition *in vivo*. The results represent further evidence that the molecular environment of LHCII has profound impact on its functional characteristics, including, among others, the susceptibility to photodamage.

## Introduction

Plants have to cope with variable light conditions – maintaining efficient light harvesting while avoiding photodamage (Li et al., 2009). Prolonged exposure to excess light causes photoinhibition, that is decrease in photosynthetic activity, followed by chlorosis – bleaching of chlorophylls (Chl) – and ultimately death. The primary site of photoinhibition is PSII (Aro et al., 1993) and the major route of PSII photoinactivation involves ROS, especially singlet oxygen (^1^O_2_), formed by the interaction of molecular oxygen with the Chl triplet (^3^Chl) states (Triantaphylides et al., 2008; Vass, 2011; Fischer et al., 2013). Most of the Chls are located in the light-harvesting antenna, including the core antenna, CP43 and CP47, and LHCII monomers and trimers (van Amerongen and Croce, 2013). However, it is believed that the antenna has negligible role in the production of ROS because the ^3^Chl states are effectively quenched by carotenoids (Cars) bound to the complexes (Breton et al., 1979; Sonneveld et al., 1979; Frank and Cogdell, 1996). In contrast, ^3^Chl states in the PSII RC are readily formed following charge recombination (Vass and Cser, 2009; Vass, 2011) and, because they are relatively far from the nearest Cars, quenching is less efficient. The formation of ^1^O_2_ during light exposure of chloroplast thylakoid membranes has been directly followed by spin-trapping EPR spectroscopy and associated with the acceptor-side inhibition of PSII and the D_1_ protein degradation (Hideg et al., 1994a, b).

Despite the abundance of Cars, ^3^Chl have been detected in isolated core antenna (Carbonera et al., 1992b; Groot et al., 1995) and peripheral antenna complexes (Carbonera et al., 1992a; Peterman et al., 1995; Barzda et al., 1998) and found to sensitize the formation of ROS, including ^1^O_2_ (Rinalducci et al., 2004). As a result, Chl PB has been observed in native and recombinant LHCII exposed to strong illumination in aerobic conditions (Formaggio et al., 2001; Zhang et al., 2008) and found to depend on the Car composition of the complex (Croce et al., 1999). Mozzo et al. (2008) studied the quenching capacity of individual Cars in LHCII and concluded that about 5% of Chl triplets are not quenched by Cars in contrast to the earlier results (Siefermann-Harms and Ninnemann, 1982; Peterman et al., 1995). Using optical magnetic resonance, Santabarbara et al. (2002a) detected ^3^Chl in thylakoid membranes generated far from the PSII RC. Together with the observed inefficiency of excitation quenching to protect from the loss of PSII activity and the blue-shifted action spectrum of photoinhibition, they proposed the involvement of weakly coupled Chls in PSII photoinhibition (Santabarbara et al., 2001b, 2002b; Santabarbara, 2006).

When exposed to light, especially in the presence of oxygen, free Chls undergo PB or photomodification by a variety of mechanisms (Bonnett et al., 1999). Cars are also sensitive to oxidative photodamage and appear to be bleached faster than Chls upon irradiation of thylakoid membranes or PSII-enriched membranes (Yamashita and Butler, 1969; Yamashita et al., 1969; Klimov et al., 1990), which in turn accelerates the PB of Chls (Santabarbara, 2006). Also, Chls absorbing at longer wavelengths are bleached before those absorbing at shorter wavelengths (Zucchelli et al., 1988; Miller and Carpentier, 1991). PB of Chl *b* occurs at a much slower rate than Chl *a* – due to fast energy transfer between them (Carpentier et al., 1986; Peterman et al., 1997). These findings point toward the role of antenna Chls in photodamage. Several studies have followed the photodamage in isolated light-harvesting complexes (Zucchelli et al., 1988; Croce et al., 1999; Formaggio et al., 2001; Olszówka et al., 2003; Zhang et al., 2008; Zubik et al., 2011) but a quantitative analysis of the kinetics and quantum yield of pigment PB and its relevance to photoinhibition is lacking.

Not only the pigments but also the apoprotein is vulnerable to degradation by ROS, in addition to the proteolytic degradation of photosynthetic proteins known to occur during photoinhibition (Li et al., 2018). Zolla and Rinalducci (2002) reported the direct photodegradation of LHCII without the involvement of proteases (Rinalducci et al., 2004). Using spin trapping EPR spectroscopy, the group detected the generation of ROS in isolated LHCII upon irradiation with visible light and correlated it with fragmentation of the polypeptide. It was also pointed out that the cleavages take place in the hydrophilic portion of the N-terminal region. On the other hand, the protein secondary structure was not affected by PB of the bound pigments (Olszówka et al., 2003). Zubik et al. (2011) also followed changes in LHCII upon exposure to strong light and postulated the photoisomerization of Cars, particularly neoxanthin.

LHCII is known to have both structural and functional flexibility (Lambrev and Akhtar, 2019). It plays a crucial role in photoprotection by NPQ. The purpose of NPQ is precisely to minimize photodamage of the system by ROS generated under excess light. The reasoning is that when LHCII is in its quenched state, i.e., singlet excitations rapidly decay via thermal deactivation, the formation of ROS and the photodamage should be reduced; however, no quantitative experimental data exists to confirm this. The switch between light-harvesting and energy-dissipating mode involves changes in the molecular and supramolecular structure of the pigment–protein complexes (Ruban, 2016). This may include aggregation or clustering of LHCII (Horton et al., 2005), which is well known to induce strong excitation quenching – both in lipid-free aggregates (Ruban and Horton, 1992, 1994) and in protein-dense reconstituted membranes (Natali et al., 2016; Crisafi and Pandit, 2017; Akhtar et al., 2019). In addition, we have observed characteristic changes in the pigment–protein and protein–protein interactions in LHCII upon changing its molecular environment – in aggregates and reconstituted membranes – some of which are not associated with NPQ (Akhtar et al., 2015). It is not clear how these changes might affect the susceptibility to photodamage.

The aim of this work is to quantify Chl PB in isolated LHCII in different molecular environments – detergent-solubilized LHCII trimers, quenched LHCII-aggregates and reconstituted membranes. The effects of intense light treatment were analyzed by absorption and CD spectroscopy, steady-state and time-resolved fluorescence, and EPR spectroscopy. One could naively presume that LHCII is more stable in the quenched aggregates but also in lipid membranes, which are closer to the native environment. For example, higher thermostability of the complex has been shown in reconstituted lipid membranes (Yang et al., 2006). On the contrary, the results presented here reveal a markedly increased oxidative PB of Chls when LHCII is in a lipid environment. Further, we estimated the rate and quantum yield of PB of Chl *a*, compared it with the yield of ROS formation detected by EPR spectroscopy and also with predictions from theoretical modeling.

## Materials and Methods

### Preparation of LHCII

LHCII trimers were purified by solubilization of PSII-enriched membrane fragments isolated from 14-days-old greenhouse grown pea (*Pisum sativum*) leaves with 0.7% *n*-dodecyl-β-maltoside (β-DDM, Cube Biotech, Germany) followed by sucrose gradient ultracentrifugation. Reconstituted membranes of LHCII and plant thylakoid lipids (lipid:protein ratio 100:1) were prepared using the protocol described previously (Akhtar et al., 2016). LHCII aggregates were prepared by removal of the detergent from suspension of solubilized complexes with polystyrene adsorbent beads (Bio-Beads SM-2 Resin, Bio-Rad). The Chl and Car contents were determined spectrophotometrically from 80% acetone extracts using molar absorption coefficients from Lichtenthaler (1987) and are presented in Supplementary Table 1.

### Photooxidation of LHCII Pigments

For the comparative photostability tests, samples were diluted to absorbance of 0.4 at the red maximum and placed in a glass cell of 1-cm optical pathlength. White light from a KL 2500 LED lamp (Schott, Germany) was used for irradiation, with incident PFD on the cuvette of 3000 μmol photons m^–2^ s^–1^ PAR. This PFD is equivalent to an average of ca. 2000 μmol photons m^–2^ s^–1^ in the whole sample volume. For testing the wavelength dependence of PB, the actinic light was passed through either a Schott FS red (630 nm) or a Schott FS blue (525 nm) filter, and the intensity was adjusted to obtain an equal fluorescence emission from the sample. A set of experiments was performed with light from a KL 1500 electronic lamp (Schott, Germany) passed through an SZS–22 glass cutoff filter (580 nm) to an incident PFD of 500 μmol photons m^–2^ s^–1^ PAR.

### Absorption, CD and Fluorescence Spectroscopy

Absorption and CD spectra were recorded using an Evolution 500 dual-beam spectrophotometer (Thermo Scientific, United States) and a J-815 (Jasco, Japan) spectropolarimeter in the visible range, at room temperature, with spectral bandwidth of 1.5 nm and 3 nm, respectively. The absorbance of the samples was 0.4 at the red maximum in a 1-cm pathlength cuvette. Synchrotron-radiation UV CD spectra were recorded at the B23 CD beamline of the Diamond synchrotron (United Kingdom). Fluorescence emission spectra in the visible range were measured from the same samples, at room temperature, on a FP-8500 (Jasco, Japan) spectrofluorometer.

### Time-Resolved Fluorescence Spectroscopy

Room temperature fluorescence decays were recorded by TCSPC using a FluoTime 200/PicoHarp 300 spectrometer (PicoQuant, Germany) as described elsewhere (Akhtar et al., 2016). A WhiteLase Micro supercontinuum fiber laser (NKT Photonics, United Kingdom) at 20 MHz repetition rate was used to generate excitation pulses. Excitation wavelength of 633 nm was selected by a monochromator, and the pulse energy was attenuated to approximately 0.1 pJ with neutral density filters. Fluorescence photons were detected by a microchannel-plate detector (R3809, Hamamatsu, Japan) and timed with 4-ps resolution. The fluorescence decays were recorded from untreated LHCII samples and after 30 min of light treatment. The samples were placed in a 1.5 mm pathlength quartz cell without further dilution. The total instrument response (IRF) width was ∼50 ps (FWHM), measured using 1% Ludox as scattering solution. The fluorescence lifetimes were determined by multiexponential fitting of the fluorescence decay kinetics combined with iterative reconvolution with the IRF. The average fluorescence lifetime was calculated as $\tau_{a\,v}=\sum_{i}a_{i}\,\tau_{i}/\sum_{i}a_{i}$.

### Electron Paramagnetic Resonance Spectroscopy

The principle of the experiments was similar to the one described by Rinalducci et al. (2004). Samples for EPR measurements were prepared under dim light and contained detergent-solubilized LHCII trimers or reconstituted LHCII membranes diluted to 0.1 mg Chl/mL in case of hydrophilic spin label and spin trap, and ca. 0.3 mg Chl/mL in case of lipophilic spin label. 5 μL sample aliquots were added to glass capillaries (with ca. 1 mm internal diameter), which were irradiated for 30 min in the EPR resonator (after tuning the instrument) with the same lamp as above, with PFD of 4800 μmol photons m^–2^ s^–1^ PAR incident on the illumination grid (front window of the resonator), directly during measurements (assuming 50% cut off by the grid and efficient reflection in the resonator (Rinalducci et al., 2004), relative PFD hitting the sample was approximately same as in the optical spectroscopy experiments). Individual scans were started at different time points of irradiation.

Singlet oxygen production upon irradiation was followed in samples with 100 mM TEMPD × H_2_O (Fischer et al., 2007). TEMPD × H_2_O traps ^1^O_2_ resulting in the 4-oxo-TEMPO, which is paramagnetic and hence detectable by EPR. Spectra of dark and light-treated blank sample (only spin trap, no LHCII) were also measured to exclude contributions from or effects by other possible sensitizers from the buffer or impurities of the spin trap.

Indirect measurement of the production of singlet oxygen and other light-induced radicals was performed by following the consumption of spin labels in irradiated samples containing 0.5 mM TEMPO – giving signal only from the aqueous phase – or 50 μM 5-SASL (spin label:lipid molar ratio = ∼2:100) – giving signal primarily originating from the hydrophobic region of the vesicles/micelles (Kóta et al., 2002). For reference EPR spectra, the stable nitroxide radicals (TEMPO and 5-SASL) were measured in buffer solution at same concentrations as in samples in dark and after 30 min light treatment.

All EPR spectra of the above nitroxide radicals (spin trap adducts and spin labels) were recorded with a Brucker ELEXSYS-II E580 X-band spectrometer at room temperature, with the following instrument settings: microwave frequency of 9.38 GHz; microwave power attenuation of 10 dB (12 dB in case of TEMPO); field modulation of 1 G (3 G in case of 5-SASL); scan range of 100 G, and conversion time of 40.96 s. To obtain the best possible signal-to-noise ratio, spectra in the dark were measured before and after illumination (after 10 min dark incubation), whereby the final spectra were averages of 20, 10, and 4 scans, for 4-oxo-TEMPO, 5-SASL and TEMPO, respectively.

In order to determine the concentration of the nitroxide radicals (the spin labels TEMPO and 5-SASL and the trapping adduct 4-oxo-TEMPO), reference spectra were recorded from samples lacking LHCII using the same instrument settings as for LHCII-containing samples but with known concentrations of spin labels (5-SASL or TEMPO). (It should be noted that the spectrum of TEMPO and 4-oxo-TEMPO are indistinguishable as concerns intensity calibration.) A linear fit to the plot of the integrated EPR absorptions (second integrals of the spectra) versus the known spin label concentrations served as a calibration to calculate nitroxide radical concentrations from the EPR spectra.

### Data Analysis

All data processing, statistical analyses and theoretical computations were done in MATLAB using the Spectr-O-Matic toolbox (available at the MATLAB File Exchange) and homebuilt routines.

## Results

### Photobleaching Kinetics

Photobleaching of Chls in LHCII in different molecular environments was observed by monitoring the changes in absorption in the course of irradiation with intense white light. Absorption spectra of LHCII solubilized with β-DDM and reconstituted lipid membranes before and after 30 min light exposure are shown in Figure 1. Upon light illumination a marked decrease in the absorption of Chl *b* and Chl *a* was observed at 652 and 675 nm, respectively, accompanied by similar changes in the Soret region. Across the visible wavelength region, the degree of PB was significantly higher in reconstituted membranes than in detergent solution (β-DDM). Qualitatively the changes are similar in all sample types (for LHCII aggregates, see Supplementary Figure 1a). As seen in the difference spectra, the Chl *a* bands at 675, 436 nm undergo the most bleaching, Chl *b* bands 652, 485 nm are less bleached and no appreciable PB of Cars is observed (450, 500 nm).

**FIGURE 1:**
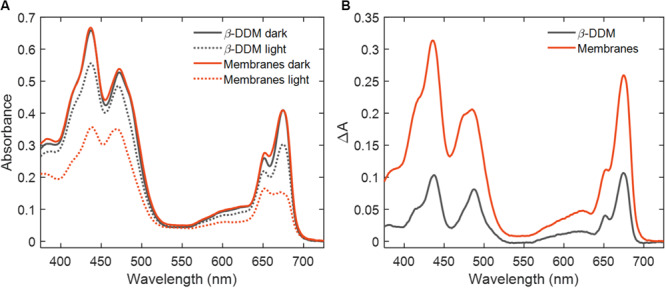
Photobleaching in LHCII. **(A)** Absorption spectra of LHCII solubilized in detergent (β-DDM) and reconstituted membranes before (solid lines) and after 30 min of irradiation (dotted lines). **(B)** Absorption difference spectra (dark–minus–irradiated sample).

**FIGURE 2:**
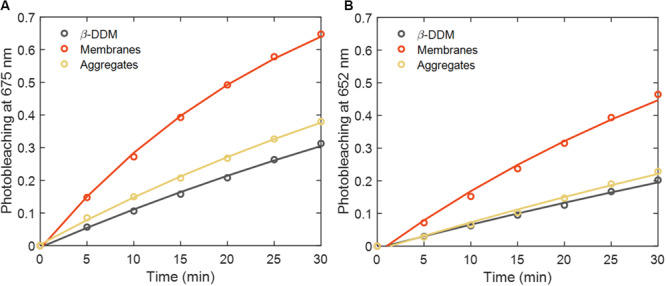
Time course of LHCII photobleaching in detergent (β-DDM), reconstituted membranes and aggregates during 30 min of irradiation (2000 μmol photons m**^–^**^2^ s**^–^**^1^). **(A)** Absorbance changes at 675 nm and **(B)** at 652 nm. Circles and lines represent experimental data points and monoexponential fits, respectively.

Figure 2 shows the degree of PB of Chls in LHCII in different molecular environments – in detergent (β-DDM), aggregates and reconstituted membranes during 30 min of irradiation. The bleaching is quantified as the relative irradiation-induced absorption difference Δ*A/A*. The time courses reinforce the finding that reconstituted membranes are significantly more susceptible to PB than either detergent-solubilized or aggregated LHCII. The transients at 675 and 652 nm, mainly associated with the Q_*y*_ transitions of Chl *a* and *b*, respectively, fit well to monoexponential kinetics, especially for the Chl *a* band (*R*^2^ > 0.99). This indicates that PB is a (pseudo) first-order process: Δ*A*/*A* = 1−*e*^−*k*_*pb*_*t*^, parametrized by the PB rate constant *k*_*pb*_ (Croce et al., 1999).

First-order PB rate constants and the respective quantum yields of PB, *φ*_*pb*_, for LHCII in different environments are shown in Table 1. The quantum yield was calculated as the ratio *φ*_*pb*_ = *k*_*pb*_/*k*_*abs*_ with *k*_*abs*_ being the absorbed photon flux per Chl (at the beginning of irradiation). The latter was estimated by integrating over the entire wavelength region, taking into account the product of the wavelength-dependent light intensity and absorption cross-section (assuming it does not vary among sample types). The quantum yield *φ*_*pb*_ in detergent-solubilized trimers is more than an order of magnitude lower than that of free Chls (Aronoff and Mackinney, 1943) but comparable to the PB of various porphyrins (Spikes, 1992; Bonnett et al., 1999). In LHCII aggregates, prepared by removing the detergent from the medium, the degree of PB was about 20% higher. Even more notably, we found that the PB yield was two- to three-fold higher in reconstituted membranes than in detergent micelles. To test whether PB in membranes is oxygen-dependent, we performed experiments in anoxic environment (continuously bubbling the reaction mixture with N_2_ gas) and in the presence of sodium ascorbate as an antioxidant. In both cases the PB was reduced to values comparable with those of detergent-solubilized LHCII (Table 1).

**TABLE 1:** Photobleaching rate constants and quantum yields for LHCII in different environments.

**LHCII environment**	**PB after 30 min **Δ***A*_675_/*A*_675_ (%)**	**PB rate constant *k*_*pb*_ (s^–1^)**	**Quantum yield *φ*_*pb*_**
β-DDM	29 ± 2*	(1.9 ± 0.1) × 10^–4^	(1.4 ± 0.1) × 10^–5^
Aggregates	34 ± 2	(2.3 ± 0.2) × 10^–4^	(1.7 ± 0.1) × 10^–5^
Reconstituted membranes	54 ± 2	(4.5 ± 0.3) × 10^–4^	(3.4 ± 0.3) × 10^–5^
Anoxic	34 ± 5	(2.3 ± 0.4) × 10^–4^	(1.7 ± 0.3) × 10^–5^
10 mM Na-ascorbate	32 ± 3	(2.1 ± 0.2) × 10^–4^	(1.6 ± 0.2) × 10^–5^
20 mM Na-ascorbate	29 ± 2	(1.8 ± 0.1) × 10^–4^	(1.3 ± 0.1) × 10^–5^

*Values represent standard error (*n* = 3–9).

A separate set of experiments was conducted on all LHCII sample types listed above with a different light treatment regime – using a tungsten halogen light source through a blue colored-glass filter and an incident PFD of 500 μmol photons m^–2^ s^–1^ (Supplementary Figure 2). Under these conditions, PB was substantially slower but qualitatively the results were similar; more importantly, *φ*_*pb*_ was comparable as with high-intensity LED irradiation (Supplementary Table 2). Further, we performed treatment with red and blue actinic light with intensities adjusted to achieve identical excitation flux. The fluorescence intensity was measured from the sample excited by either red or blue light to confirm the equal absorbed photon flux. The PB rate was identical in both cases (Supplementary Figure 3), therefore *φ*_*pb*_ is wavelength-independent.

### CD Spectral Changes

**FIGURE 3:**
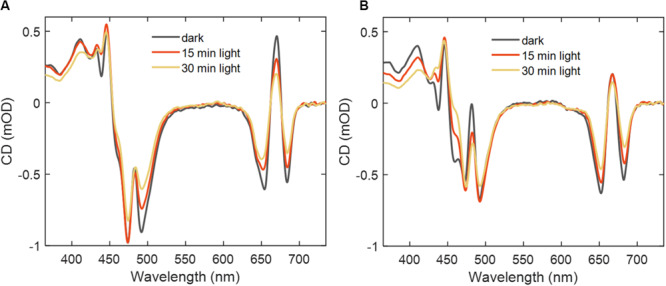
CD spectra of LHCII before, and after 15 and 30 min of irradiation. **(A)** LHCII in detergent (β-DDM) and **(B)** in reconstituted membranes. The spectra correspond to absorbance 0.4 at 675 nm.

We employed CD spectroscopy to monitor the structural/conformational changes in LHCII induced by intense irradiation. The CD spectra of complexes in detergent and reconstituted membranes (Figure 3) show significant changes both in the Soret as well as the Chl *Q*_*y*_ region; the same applies for LHCII aggregates (Supplementary Figure 1b). The CD amplitude in the Chl *Q*_*y*_ region decreased proportionally to the decrease in the absorption (PB) and the shape of the spectra remained unchanged – indicating that the general structure of the pigment–protein complex remains intact even though a large part of the chromophores are lost (Olszówka et al., 2003). In the Soret region, there were additional changes – especially at 494 nm in β-DDM – which were not only caused by the loss of absorbance. This is better illustrated in the spectra of the CD/A ratio (Supplementary Figure 4). Significant loss of CD amplitude at these wavelengths occurred already after 15 min of irradiation. The changes could be due to a disruption of excitonic couplings between (Chl and Car) transitions in the blue wavelength range or due to changes in the induced CD of Cars. The negative CD bands at 438 and 460 nm in reconstituted membranes and aggregates, which are associated with inter-trimer interactions, rapidly diminished upon irradiation.

### Fluorescence Quenching

**FIGURE 4:**
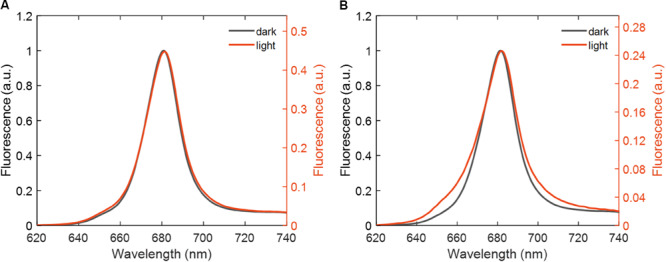
Fluorescence emission spectra of LHCII before and after 30 min irradiation recorded with 436 nm excitation light. **(A)** LHCII in detergent (β-DDM) and **(B)** in reconstituted membranes. Note the separate intensity axes (right side) for irradiated samples.

Room-temperature fluorescence emission spectra of LHCII in detergent micelles and reconstituted membranes recorded with 436 nm excitation before and after 30 min of irradiation are shown in Figure 4. The fluorescence emission was strongly reduced compared to the unexposed samples. Even after correcting for the loss of absorption at the excitation wavelength, the fluorescence yield was reduced by a factor of 2.2 in detergent-solubilized LHCII and 5–7 in reconstituted membranes and aggregates. In all sample types, the degree of fluorescence quenching substantially exceeded the PB (loss of absorption), suggesting that irradiation induced non-radiative dissipation in the partially photobleached complexes. Some spectral changes can also be noted. The relative fluorescence intensity in the Chl *b* region (650–660 nm) was enhanced, especially in reconstituted membranes. The shape and width of the main emission band at 680 nm remained almost the same, except for a slight (∼1 nm) red shift of the maximum. The normalized spectra of irradiated reconstituted membranes (Figure 4B) as well as aggregates (Supplementary Figure 5a) showed enhanced fluorescence emission in the far-red region (700–720 nm).

Further we performed picosecond time-resolved fluorescence measurements of the LHCII samples by TCSPC. The fluorescence recorded at 680 nm after 30 min of irradiation showed an initial phase of rapid decay in all the tested environments (Supplementary Figures 5b, 6), confirming the light-induced quenching observed by steady-state fluorescence. For a quantitative analysis, the fluorescence decay curves were subjected to multiexponential fitting. The resultant decay lifetimes, their relative amplitudes and the average fluorescence lifetimes at 680 nm are shown in Table 2. The average lifetime τ_*av*_ = $\sum_{i}a_{i}$$\tau_{i}/\sum_{i}a_{i}$ decreased by a factor of 2.3 for detergent-solubilized LHCII, in good agreement with the steady-state fluorescence data, and by a factor of 3–4 for reconstituted membranes and aggregates. The somewhat lower quenching factors estimated from time-resolved fluorescence suggest the presence of fast decay components that are below the time resolution of the measurement.

**TABLE 2:** Fluorescence lifetime analysis of LHCII in different environments.

**Sample**	**Irradiation**	***τ*_1_ (ns)**	***a*_1_ (%)**	***τ*_2_ (ns)**	***a*_2_ (%)**	***τ*_3_ (ns)**	***a*_3_ (%)**	***τ*_4_ (ns)**	***a*_4_ (%)**	***τ*_*av*_ (ns)**
β-DDM	–					0.8	5	3.8	95	3.6
	30 min	0.07	27	0.27	21	1.3	16	3.6	35	1.6
Membranes	–			0.30	16	1.1	60	2.9	24	1.4
	30 min	0.08	44	0.28	35	0.9	17	2.5	4	0.4
Aggregates	–	0.11	61	0.33	33	1.0	6	3.1	1	0.26
	30 min	0.06	81	0.19	18	0.6	2	3.0	0.1	0.10

The fluorescence of LHCII in detergent decayed almost monoexponentially, as it is well known, with a lifetime of 3.8 ns and a very small (5%) contribution from a shorter, 0.8-ns component. After 30 min irradiation, at least two additional shorter decay lifetimes were observed – about 70 and 300 ps – with a combined amplitude of approximately 50%. Similar decay lifetimes (80 and 300 ps) appeared after irradiation of LHCII in reconstituted membranes, in this case having a combined amplitude of 80%, at the expense of the nanosecond decay components. In irradiated aggregates, 80% of the excitations decayed with a lifetime of 60 ps. The absence of blue-shifted emission components in the decay-associated spectra (data not shown) and long lifetimes shows that no free/uncoupled Chls were present in the irradiated samples.

### Electron Paramagnetic Resonance

**FIGURE 5:**
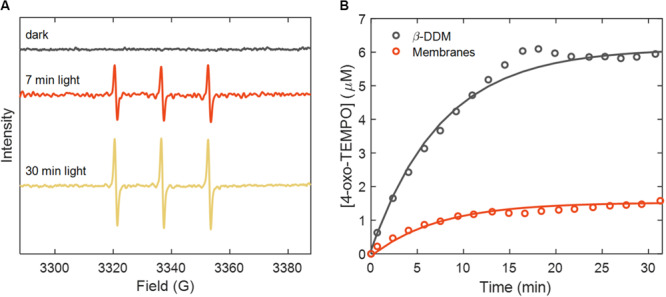
EPR spectra of 4-oxo-TEMPO, generated during illumination of LHCII membranes in the presence of 100 mM 4-oxo-TEMP (TEMPD × H_2_O). **(A)** Spectra recorded before and after 7 and 30 min of irradiation of reconstituted membranes. **(B)** Time course of singlet oxygen trapping by 4-oxo-TEMP (TEMPD × H_2_O) during 30 min irradiation. Circles and lines represent experimental data points and monoexponential fits, respectively.

To identify and quantify the ROS formed during irradiation of LHCII, EPR measurements were performed at different intervals after light exposure of samples containing either the spin trap TEMPD or the spin labels TEMPO and 5-SASL. The EPR-silent spin trap 4-oxo-TEMP (TEMPD) converts to the paramagnetic nitroxide radical 4-oxo-TEMPO upon reaction with ^1^O_2_ (Lion et al., 1976), yielding a specific EPR spectrum almost identical to that of the TEMPO spin label (Marshall et al., 2011). A dose-dependent EPR signal, typical for 4-oxo-TEMPO, was detected after irradiation of LHCII-containing samples (Figure 5A). No signal was observed in samples kept in the dark or after illumination of the spin-label-containing buffer/liposomes without LHCII (data not shown).

The dependence of the 4-oxo-TEMPO concentration on illumination time, estimated from the intensity of the central EPR band, is plotted in Figure 5B, along with exponential fits for LHCII in reconstituted membranes and β-DDM. The 4-oxo-TEMPO signal nearly saturated after 30 min irradiation and the total detected concentration was about 4-fold higher (6 μM) in detergent-solubilized LHCII than in reconstituted membranes (1.5 μM). On the other hand, for the initial exponential phase of the curves, the fitted time constant of radical formation was shorter for membranes than detergent (5 vs. 10 min) – thus the initial rate of ^1^O_2_ generation was higher in reconstituted membranes.

**FIGURE 6:**
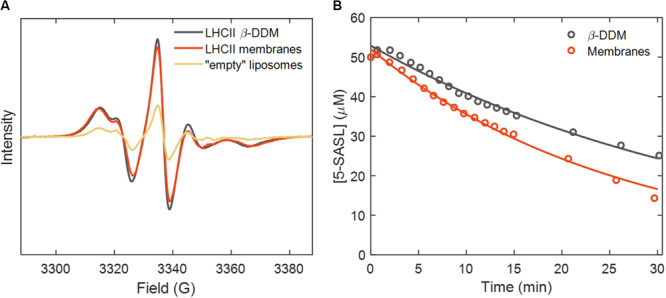
**(A)** EPR spectra of 5-SASL in detergent micelles and lipid membranes. **(B)** Time course of the 5-SASL concentration estimated from the EPR signal intensity during 30 min of irradiation of the reaction mixture containing LHCII with 50 μM 5-SASL. Circles and lines represent experimental data points and monoexponential fits, respectively.

Figure 6 shows the EPR spectra and illumination time course of samples containing 5-SASL. Stearic acid spin labels, such as 5-SASL, partition between the membrane and the aqueous buffer with very high preference toward membranes. The spectra of thylakoid lipid vesicles, LHCII proteoliposomes as well as of detergent-solubilized LHCII all showed features typical for 5-SASL in membrane phase (see, e.g., Kóta et al., 2002; Páli and Kóta, 2019), whereas the aqueous-phase EPR signature was less than 1% and could be neglected. 5-SASL can react with various radicals thereby losing its EPR signal, via either one electron oxidation or reduction. As Figure 6B shows, after the onset of illumination, the 5-SASL concentration decayed approximately exponentially in both LHCII-containing samples but the rate of quenching was approximately double in reconstituted membranes than in detergent solution. The same result was obtained when using TEMPO instead of 5-SASL (Supplementary Figure 7).

## Discussion

### The Degree of PB Does Not Correlate With the Excited-State Lifetime

The investigation of Chl PB in LHCII presented here confirms earlier observations that the light-harvesting antenna complexes are sensitive to photodamage when they are not coupled to active RCs (Siefermann-Harms, 1990; Zolla and Rinalducci, 2002; Rinalducci et al., 2004; Zhang et al., 2008). The quantum yield of Chl PB, *φ*_*pb*_, ranges between 1 × 10^–5^ and 4 × 10^–5^. A key experimental result is the non-trivial dependence of *φ*_*pb*_ on the molecular environment of LHCII. Firstly, higher-order aggregation of LHCII trimers leads to effective quenching of singlet excited states, diminishing the singlet lifetime by a factor of 20 in accord with numerous studies (Horton et al., 1991; Mullineaux et al., 1993; Miloslavina et al., 2008). One would expect a proportional reduction in *φ*_*pb*_, assuming that the photosensitizer agent is Chl^∗^. The results, however, show that *φ*_*pb*_ is slightly increased instead. In this sense LHCII aggregation, which is considered as a model of NPQ, does not seem to have a photoprotective effect on LHCII itself, although evidently any quenching mechanism will relieve the excitation pressure on PSII and have a photoprotective role *in vivo*. Lack of expected correlation between the excited-state lifetime and photoinactivation has also been noticed in thylakoid membranes. The loss of PSII activity or D1 degradation showed no or only mild correlation with the excitation quenching induced by spillover or the addition of exogeneous quenchers (Tyystjärvi et al., 1999; Santabarbara et al., 2001a, 2002a). Exogeneous quenchers exerted a modest protection from PB of Chls in light-exposed thylakoids (Santabarbara, 2006). The dose response of both photoinhibition and PB, the lack of a linear relationship, and the blue-shifted action spectrum of photoinhibition led to the suggestion that a small population of antenna complexes in which Chl–Car coupling is impaired, are mainly responsible for ROS generation and photodamage in the thylakoid membranes (Santabarbara et al., 2001b; Santabarbara, 2006). In the following sections we focus on the formation of ^3^Chl and their quenching by Cars in LHCII.

Perhaps the most striking result of the current investigation is that LHCII in reconstituted membranes is significantly (nearly three-fold) more sensitive to photodamage than either LHCII aggregates or detergent-solubilized trimers. To understand these results, it is demanding to comprehend the specific photochemical mechanisms of photodamage.

### Fluorescence Quenchers Are Generated in the Course of PB

Both steady-state and time-resolved fluorescence measurements revealed that the Chl fluorescence yield *φ*_*F*_ and lifetime τ_*F*_ are significantly reduced upon irradiation in all types of samples, indicating that PB is associated with the generation of quenchers. Light-induced fluorescence quenching has been known to occur in isolated LHCII and especially in lamellar LHCII aggregates (Jennings et al., 1991; Barzda et al., 1996). Quenching in irradiated LHCII liposomes has been reported by Zubik et al. (2011), who ascribed it to Car photoisomerization and formation of long-lived quencher states, particularly Chl–Car charge-transfer states, owing to the increased absorption and Stark effect around 900 nm. These results are consistent with a more general interpretation that the photoproducts, be it long-lived Chl radicals or other derivates, possibly bilinone analogs (Jose et al., 1990), may act as fluorescence quenchers in the photodamaged complexes. Upon prolonged irradiation this fluorescence quenching might have a self-protecting role; however, further quantitative analysis would be necessary to test this.

### Photobleaching Is Caused by Singlet Oxygen Produced by Chl Triplets

Chlorophyll PB in LHCII in reconstituted membranes was effectively suppressed in anaerobic environment (Table 1) or by adding ascorbate in aerobic conditions, as has been shown for isolated LHCII (Siefermann-Harms, 1990; Croce et al., 1999), confirming that it is, for the main part, oxidative. The quantum yield *φ*_*pb*_ was independent of the intensity and wavelength of the actinic light (Supplementary Figures 2, 3 and Supplementary Table 2), indicating that the reaction is one-photon and initiated by the lowest-lying singlet-excited state of Chl. Moreover, very little PB of Chl *b* was observed, which is consistent with results on solubilized LHCII (Croce et al., 1999; Olszówka et al., 2003; Zhang et al., 2008) and the fact that Chl *b* transfers energy to Chl *a* on a much shorter timescale than the formation of triplets (Connelly et al., 1997). All these data corroborate that the PB occurs via a type II reaction photosensitized by Chl triplet states (^3^Chl), which has been thoroughly demonstrated for Chls (Krasnovskii Jr., 1994). Moreover, the sensitizer is Chl *a* as Chl *b* triplets have not been detected in LHCII (Peterman et al., 1997). Presumably, the triplet Chl *a* reacts with molecular oxygen producing singlet oxygen (^1^O_2_) which then attacks the Chl directly or is transformed to another ROS, e.g., a hydroxyl radical.

The formation of ^1^O_2_ in reconstituted LHCII membranes was confirmed directly and indirectly by EPR in agreement with the experiments of Zolla and Rinalducci (2002) and Rinalducci et al. (2004). The spin trap 4-oxo-TEMP, directly sensing ^1^O_2_, produced 4-oxo-TEMPO radicals only in irradiated samples containing Chl. In principle, the EPR analysis is quantitative, meaning that we should be able to estimate the ^1^O_2_ yield from the time-dependent concentration of the spin labels. However, the hydrate form of 4-oxo-TEMP (TEMPD × H_2_O) is water-soluble and partitioned entirely in the aqueous phase whereas ^1^O_2_ is produced in the hydrophobic lipid/protein phase. The 4-oxo-TEMPO concentration then depends not only on the rate of ^1^O_2_ formation but also on its solubility, diffusion and lifetime in the different phases. For this reason, quantifying the ^1^O_2_ yield in different environments is not straightforward. The reaction mixture of LHCII–lipid membranes contains substantial amount of lipids (0.9 mM). The lipids are at the same time solvent for the oxygen and substrate for lipid peroxidation, which may explain why 4-oxo-TEMP reported less overall amount of ^1^O_2_ in the reconstituted membranes than in detergent LHCII. On the other hand, the initial rate of 4-oxo-TEMPO formation was higher in reconstituted membranes but the signal saturated at a lower level. This is probably because of heterogeneity of the sample with only a fraction of the LHCII complexes exposed to the spin trap, e.g., those on the outer sheet of multilamellar vesicles.

In contrast to 4-oxo-TEMP, the EPR spectrum of the spin label 5-SASL evidenced its incorporation into the lipid phase (Kóta et al., 2002; Páli and Kóta, 2019) which is the site where ROS are formed (we observe negligible aqueous 5-SASL signal, with sharp lines). In principle, the loss of 5-SASL EPR intensity over time should reflect the ROS produced during irradiation of the samples – 0.06 and 0.11 mol/mol Chl for LHCII in detergent and membranes, respectively, after 30 min. With a large excess of free spin label, we can approximate the kinetics to be first-order with rate constants of 4.3 × 10^–4^ s^–1^ and 6.3 × 10^–4^ s^–1^, respectively. These values correspond to quantum yields of radical formation of 2 × 10^–5^ (β-DDM) and 4 × 10^–5^ (lipid membranes), comparing well with *φ*_*pb*_. However, these values must also be taken with caution because in detergent micelles, a large fraction of 5-SASL must be incorporated in micelles that do not contain any LHCII, whereas the majority of lipid vesicles contain more than one LHCII trimer (Tutkus et al., 2018). For a more accurate modeling of the ROS dynamics in such a heterogeneous system, the partitioning and mobility of both the ROS and the spin probe in all phases must be accounted for.

### A Kinetic Model of Singlet Oxygen Formation

The rate of the photosensitization reaction is proportional to the concentration of ^3^Chl states, which in turn depends on the ability of Cars in LHCII to quench ^3^Chls. Several studies have shown that the triplet–triplet (T–T) energy transfer from Chls to Cars in LHCII occurs with near 100% efficiency (Siefermann-Harms and Ninnemann, 1982; Peterman et al., 1995) – which is the very reason why antenna PB should be negligible in the first place. Even if that is the case, low transient concentration of ^3^Chl may still generate ^1^O_2_. To address this question quantitatively, having in mind the considerations above, we can construct a simplified kinetic model of the PB reaction (Scheme 1). The relevant kinetic parameters are the rate constants of Chl singlet and triplet decay, *k*_*D*_ and *k*_*T*_, intersystem crossing, *k*_*ISC*_, T–T transfer to Cars, *k*_*T–T*_, the sensitization rate constant *k*_*ox*_ and the local oxygen concentration [O_2_]. The ^3^Chl yield φ_*T*_ can be calculated as

**FIGURE 1:**
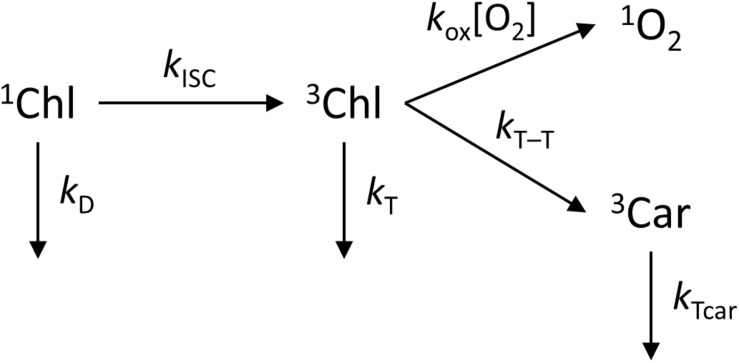
Kinetic scheme of the photosensitization of singlet oxygen in LHCII.

$$\varphi_{T}=\frac{k_{I\,S\,C}}{k_{D}+k_{I\,S\,C}}.$$

The rate constant *k*_*ISC*_ for Chl *a* is 0.1 ns^–1^ (Bowers and Porter, 1967) and the denominator is equal to the inverse fluorescence lifetime – (3.8 ns)^–1^ for detergent-solubilized LHCII (Table 2). The triplet yield is then φ_*T*_ = 0.38. The ^1^O_2_ yield is in turn given by the expression

$$\varphi_{o\,x}=\varphi_{T}\,\frac{k_{o\,x}\,\left[O_{2}\right]}{k_{o\,x}\,\left[O_{2}\right]+k_{T}+k_{T-T}}.$$

In anaerobic environment, *k*_*T*_, which is equal to the inverse triplet lifetime, *k*_*T*_ = 1/τ_*T*_, is vanishingly small, in the range of 1–2.5 ms^–1^ (Peterman et al., 1997; Niedzwiedzki and Blankenship, 2010). The Car quenching rate constant *k*_*T–T*_ has been estimated in the range of 2–10 ns^–1^ (Schödel et al., 1998). Finally, for estimating *k*_*ox*_ we refer to measurements on free Chl, where we can make use of the relation

$$\tau_{T}=\frac{1}{k_{T}+k_{o\,x}\,\left[{\text{O}}_{2}\right]}.$$

In aerated organic solvents *τ*_*T*_ is about 0.3 μs (Drzewiecka-Matuszek et al., 2005; Niedzwiedzki et al., 2014). Consequently, *k*_*ox*_ ≈ 2 × 10^9^ M^–1^ s^–1^ (calculating the oxygen concentration in water at 20°C to be 1.4 mM), which is consistent with the values reported for Chl *a* and its derivatives (Mathis and Kleo, 1973; Fiedor et al., 1993).

Using these rate parameters and the equations above, we calculate values of *φ*_*ox*_ in the range of 1 × 10^–4^ to 5 × 10^–4^. The time dependence of ^3^Chl and ^1^O_2_ upon Chl excitation, obtained by solving the kinetic model, is given in Supplementary Figure 8. The theoretically estimated quantum yield is comparable to the yield of ROS formation reported by EPR (see above). Therefore, the calculations demonstrate that the transient concentration of ^3^Chl, which decays with the singlet excitation lifetime, is in principle sufficient to generate ample amounts of ^1^O_2_ to account for the observed PB (*φ*_*pb*_ ≈ 10^–5^). The calculations also show unequivocally that *φ*_*ox*_, and therefore PB, should be linearly proportional to the fluorescence lifetime. Thus, the rate of PB in reconstituted LHCII membranes and in aggregates should be 3-fold and 20-fold lower, respectively, than in detergent micelles (all other parameters being constant).

### Why Is LHCII More Susceptible to PB in Lipid Membranes?

The observed variation of *φ*_*pb*_ with the molecular environment can be explained with variations in either the rates *k*_*ISC*_, *k*_*T*__–__*T*_, or *k*_*ox*_[O_2_] in the kinetic model discussed above. In principle, all of these are possible. Both *φ*_*T*_ and τ_*T*_ are shown to depend on the solvent environment (Hurley et al., 1980). The quenching of ^3^Chl by Cars strongly depends on the arrangement of the pigments in the complex, with an exponential dependence on the distance between them, stemming from the Dexter exchange transfer mechanism (Siefermann-Harms, 1987). Comparatively small structural alterations, for example induced by increasing the detergent concentration, can affect the energetic coupling between pigments, reducing *k*_*T*__–__*T*_, and in turn raising the ^3^Chl yield (Naqvi et al., 1999). The CD spectra of LHCII, especially in the Cars region, indicate that such conformational changes occur upon aggregation and in the lipid environment (Akhtar et al., 2015). From the kinetic model it follows that a two-fold reduction in *k*_*T*__–__*T*_ will result in a corresponding two-fold increase in *φ*_*ox*_. It may also be speculated that some Cars are destabilized or missing in the artificial reconstituted membranes; however, this seems unlikely because we do not detect a significant change in the pigment composition (Supplementary Table 1). Moreover, the primary quenchers of the terminal emitter Chl *a* are luteins (Dall’Osto et al., 2006) and their loss would result in protein unfolding (Formaggio et al., 2001), which has not been detected in the UV-CD of irradiated samples in our experiments (data not shown) or in previous studies (Olszówka et al., 2003). While photodegradation of the LHCII apoprotein does occur, it only involves the N-terminus (Zolla and Rinalducci, 2002).

Another potential factor affecting *φ*_*ox*_, and hence *φ*_*pb*_ is the local O_2_ concentration or the O_2_ accessibility to the site of ^3^Chl formation. This may well be a leading cause for the enhanced photosensitivity of LHCII in lipid membranes, as it has been shown that the lipid/water partitioning factor of O_2_ in phospholipid liposomes and lipoproteins is up to 4, particularly in the liquid crystalline phase (Möller et al., 2005, 2016). If the O_2_ concentration is higher in the vicinity of the LHCII pigments, that will also affect the Chl and Car triplet lifetimes. Careful comparison of the triplet lifetimes in LHCII in different environments might be useful to test this hypothesis.

Finally, we must consider that PB may also be indirectly caused by ROS generated in a radical chain reaction, for example by alkoxyl radicals. In that case, lipids and lipid peroxidation products may act as secondary sensitizers for the PB. In support of this, we have been able to detect, although semi-quantitatively, lipid peroxidation products in light-exposed LHCII liposomes via a malondialdehyde–thiobarbituric acid reactivity assay (data not shown). However, we did not observe significant photoprotective effect of adding α-tocopherol to the reaction mixture, in agreement with results on solubilized LHCII (Siefermann-Harms, 1990), suggesting that alcoxyl radicals are not a dominant trigger of Chl PB in the liposomes. Whether lipid peroxidation is actually involved in the photodegradation of Chls is purely a speculation but in either case our results point to an intrinsic volatility of the lipid environment with respect to photodamage that must not be overlooked. Neither the local O_2_ concentration, nor lipid peroxidation readily explain the differences, or lack thereof, between the Chl PB in detergent-solubilized and aggregated LHCII. Therefore, we tend to assume that the PB dependency on the environment is due to a combination of several factors discussed above.

### Is Chl PB in LHCII Relevant to Photoinhibition *in vivo*?

So far it remains unclear whether direct photodegradation of the antenna has a significant role in photoinhibition *in vivo*. In comparison to the values of *φ*_*pb*_ obtained here, photoinhibition of PSII occurs with a significantly lower quantum yield, in the order of 10^–7^ (Campbell and Tyystjärvi, 2012). In active PSII, LHCII excitations are rapidly transferred to the RC and quenched by photochemistry, so the PB in the functionally connected antenna will be far less than in the isolated complexes. However, if the RCs are closed, which can be the case under prolonged excess light conditions, this photoprotective route is unavailable (Lambrev et al., 2012; Ruban, 2016). Then, considering that ∼10^2^ antenna Chls are connected to one PSII RC, we can expect that the rates of direct PB of antenna Chls and PSII photoinhibition will have the same order of magnitude. In native thylakoids subjected to photoinhibitory treatment, the rate of ^1^O_2_ production declined by about half after the complete loss of oxygen evolution (Hideg et al., 1994b). Taken together with a report of no appreciable formation of ^1^O_2_ by Photosystem I (Hideg and Vass, 1995), this invites the hypothesis that the excess ^1^O_2_ is produced by the PSII antenna. However, the same authors found no ^1^O_2_ formation in thylakoids upon donor-side inactivation of PSII electron transport (Hideg et al., 1994a), which either invalidates the antenna hypothesis, or it must be assumed that the oxidized RC radical, P_680_^+^, prevents the accumulation of ^1^O_2_ (for example via efficient quenching).

Pigment PB associated with photoinhibition in thylakoid membranes and PSII-enriched membranes has been experimentally shown in several studies (Yamashita and Butler, 1969; Yamashita et al., 1969; Carpentier et al., 1986; Zucchelli et al., 1988; Miller and Carpentier, 1991). The PB kinetics and the sensitivity of the different pigment pools, however, appear to be markedly different in these systems compared to isolated antenna complexes. In thylakoid membranes, Cars were found to be the primary target of the photooxidation reactions (Yamashita and Butler, 1969; Yamashita et al., 1969). Cars were also the main photobleached pigments in PSII-enriched membranes lacking manganese (Klimov et al., 1990) and in the D_1_–D_2_–Cyt b_559_ complex (Telfer et al., 1991). In a more recent study, Santabarbara (2006) found that PB in thylakoid membranes occurred in two distinct phases – a slow initial phase, during which Cars were bleached at a rate three times higher than Chl *a*, followed by a second phase marked by rapid PB of Chls – evidently because the protective role of Cars was eliminated. These results indicate that Chl PB is a late event in photoinhibition in native thylakoid membranes and a consequence of the disruption of T–T transfer from Chl to Cars. As we do not observe substantial bleaching of Cars in isolated LHCII, similar to other results (Siefermann-Harms, 1990), it could then be postulated that T–T transfer is disrupted in the isolated antenna complexes, making the Chls more susceptible to PB (Naqvi et al., 1999). According to the kinetic model, isolated LHCII should be capable of producing enough ^1^O_2_ to explain the observed Chl PB. Obviously, there is no guarantee that the model holds *in vivo*, which again exemplifies the caveats of *in vitro* experiments with a flexible protein complex such as LHCII, which is sensitive to its molecular environment (Akhtar et al., 2015).

## Conclusion

In this work, we have shown that light exposure of isolated LHCII causes oxidative PB of Chl *a* with a quantum yield of 1 × 10^–5^ to 4 × 10^–5^, which indicates that in excess light conditions, when the PSII RCs are predominantly closed, direct photodamage of the antenna could occur with rates comparable to the PSII RC photoinactivation. The sensitivity to photodamage depends on the molecular environment of the complex, such that PB is significantly exacerbated in reconstituted lipid membranes. Quantitative EPR spectroscopy analysis using spin labels confirms the increased light-induced generation of ^1^O_2_ in the membranes. This is probably a combined effect of the solubility and diffusion of oxygen and other factors modifying the ultimate fate of the excitation energy. Regardless of what the exact underlying cause is, the increased PB susceptibility of LHCII in lipid membranes is potentially of great significance considering that this is the native environment for the majority of photosynthetic pigment–protein complexes. As direct photosensitization of ROS by the light-harvesting complexes is not negligible, ROS must be effectively scavenged in the membrane to avoid photodamage.

## Data Availability Statement

The generated datasets for this study (absorption, CD, fluorescence and EPR spectra) can be found online in the Mendeley Data repository (Lingvay et al., 2020).

## Author Contributions

PL designed the experiments. ML and PA isolated LHCII, prepared reconstituted membranes, and performed optical spectroscopy measurements. ML, KS-N, and TP performed EPR measurements. ML performed data analysis. PA and PL did theoretical modeling. The manuscript was written through contributions of all authors. All authors have given approval to the final version of the manuscript.

## Conflict of Interest

The authors declare that the research was conducted in the absence of any commercial or financial relationships that could be construed as a potential conflict of interest.

## Abbreviations

^1^O_2_: singlet oxygen
4-oxo-TEMPO: (4-oxo-2,2,6,6-tetramethylpiperidin-1-yl)oxyl
5-SASL: 5-doxyl-stearic acid spin label
Car: carotenoid
CD: circular dichroism
Chl: chlorophyll
EPR: electron paramagnetic resonance
LHCII: light-harvesting complex II
NPQ: non-photochemical quenching
PAR: photosynthetically active radiation
PB: photobleaching
PFD: photon flux density
PSII: photosystem II
RC: reaction center
ROS: reactive oxygen species
TCSPC: time-correlated single-photon counting
TEMPD × H_2_O: 2,2,6,6-tetramethyl-4-piperidone monohydrate
TEMPO: (2,2,6,6-tetramethylpiperidin-1-yl)oxyl
β -DDM: *n*-dodecyl- β -maltoside.
