# Supplementary material for: Photobleaching of Chlorophyll in Light-Harvesting Complex II Increases in Lipid Environment
Source: Front Plant Sci. 2020 Jun 24;11:849. doi: 10.3389/fpls.2020.00849 (PMC7327537; doi:10.3389/fpls.2020.00849)
Supplement: Supplementary file 1 [file Data_Sheet_1.PDF]

Supplementary Material

1 Supplementary Figures and Tables

1.1 Supplementary Figures

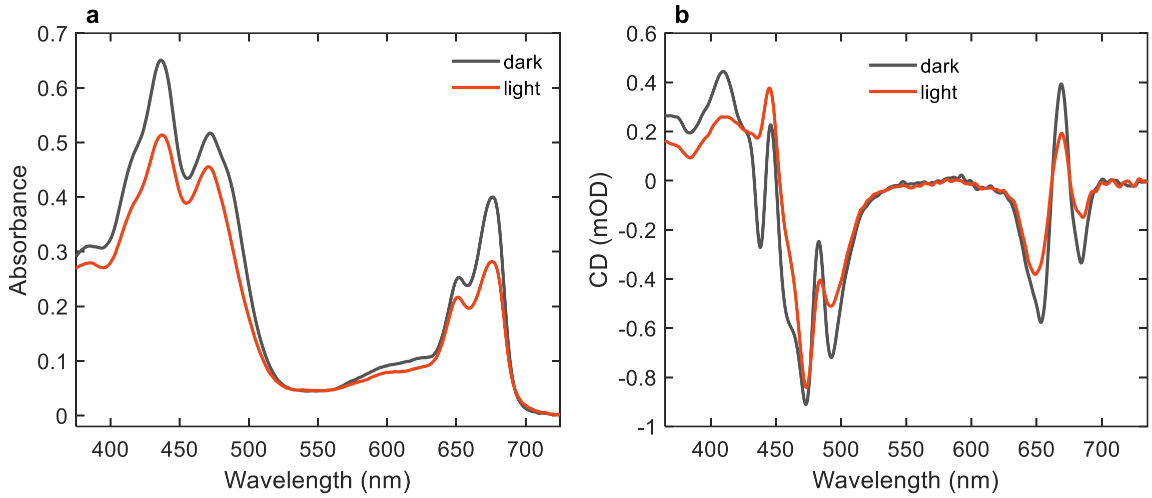

Supplementary Figure 1. Photobleaching and CD changes in LHCII aggregates. **(a)** Absorption and **(b)** CD spectra before and after 30 min of irradiation. The spectra correspond to absorbance 0.4 at 675 nm.

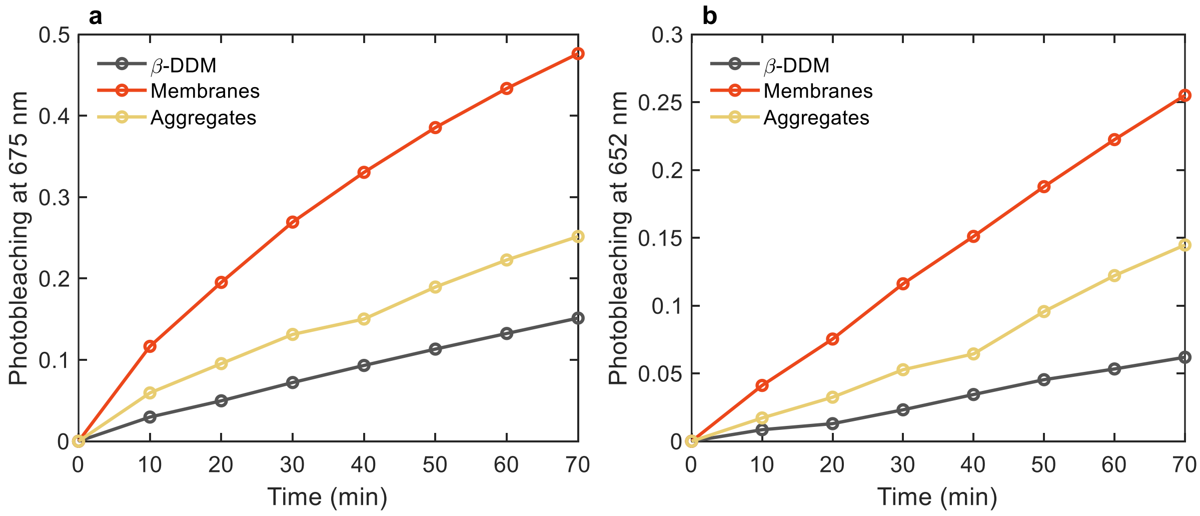

Supplementary Figure 2. Time course of LHCII photobleaching in detergent (β-DDM), reconstituted membranes and aggregates during 30 min of irradiation. The light intensity was set to 500 μmol photons m^{-2} s^{-1} through a blue colored glass cutoff filter (SZS–22, 580 nm). **(a)** Absorbance changes at 675 nm and **(b)** at 652 nm.

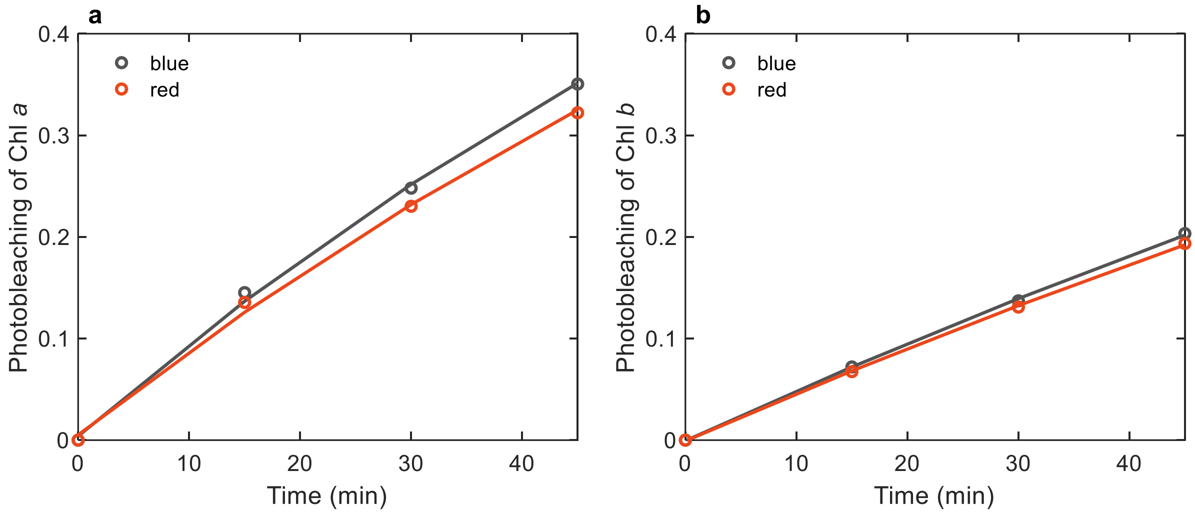

Supplementary Figure 3. Time course of LHCII photobleaching in reconstituted membranes during 45 min of irradiation. The samples were irradiated with red – Schott FS red insert filter (630 nm) – and blue – Schott FS blue insert filter (525 nm) – actinic light with intensities adjusted to achieve identical excitation flux. **(a)** Absorbance changes of Chl *a* peak and **(b)** Chl *b* peak.

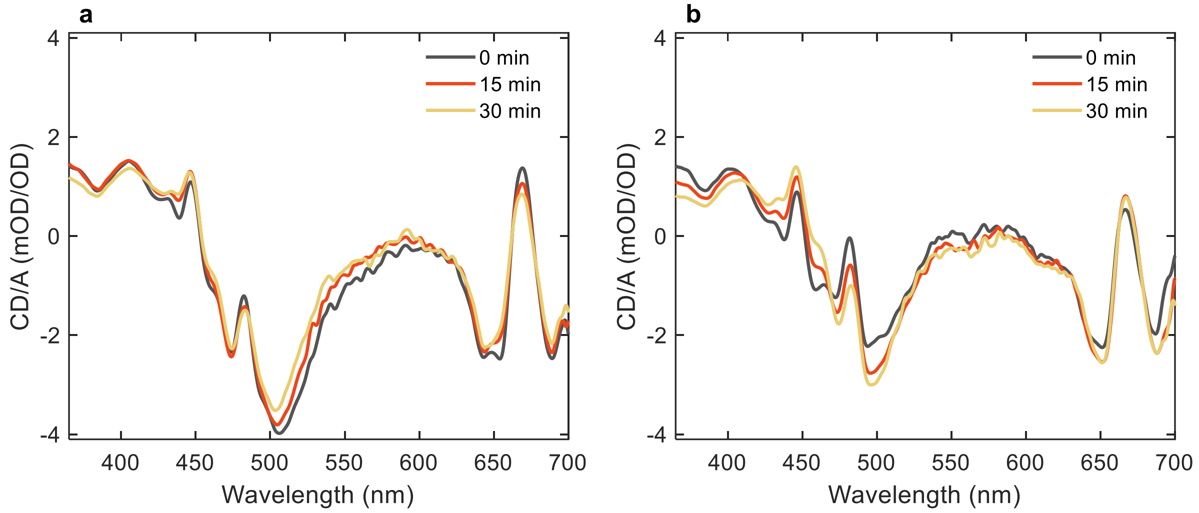

Supplementary Figure 4. CD spectra normalized to absorption spectra, before and after 15 and 30 min of irradiation. **(a)** LHCII in detergent (β-DDM) and **(b)** in reconstituted membranes.

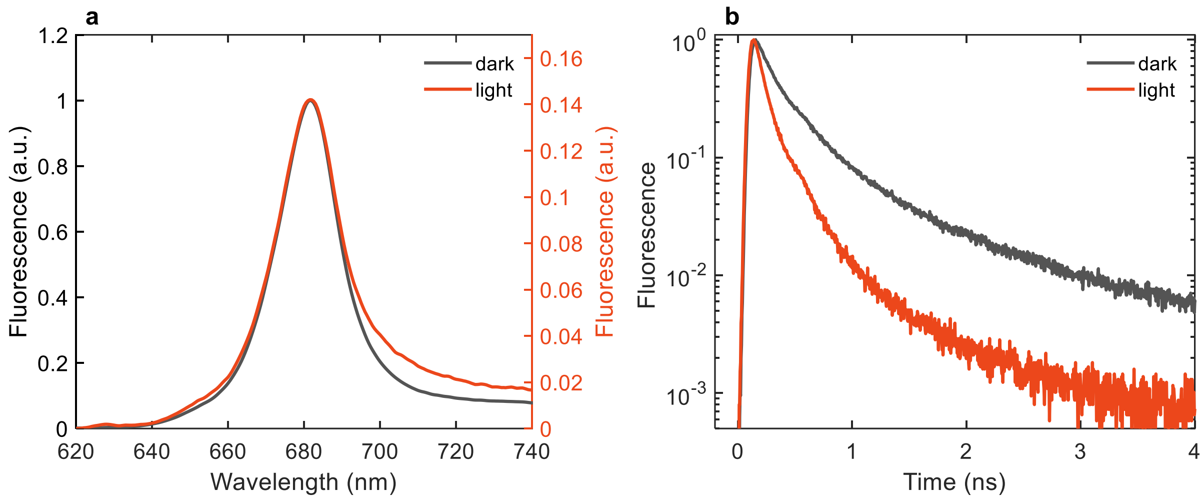

Supplementary Figure 5. Light-induced fluorescence quenching in LHCII aggregates. **(a)** Steady state fluorescence emission spectra recorded with 436 nm excitation light in the dark and after 30 min of light treatment. Note the separate intensity axes (right side) for irradiated sample. **(b)** Fluorescence decay trace at 680 nm emission wavelength before and after 30 min of irradiation.

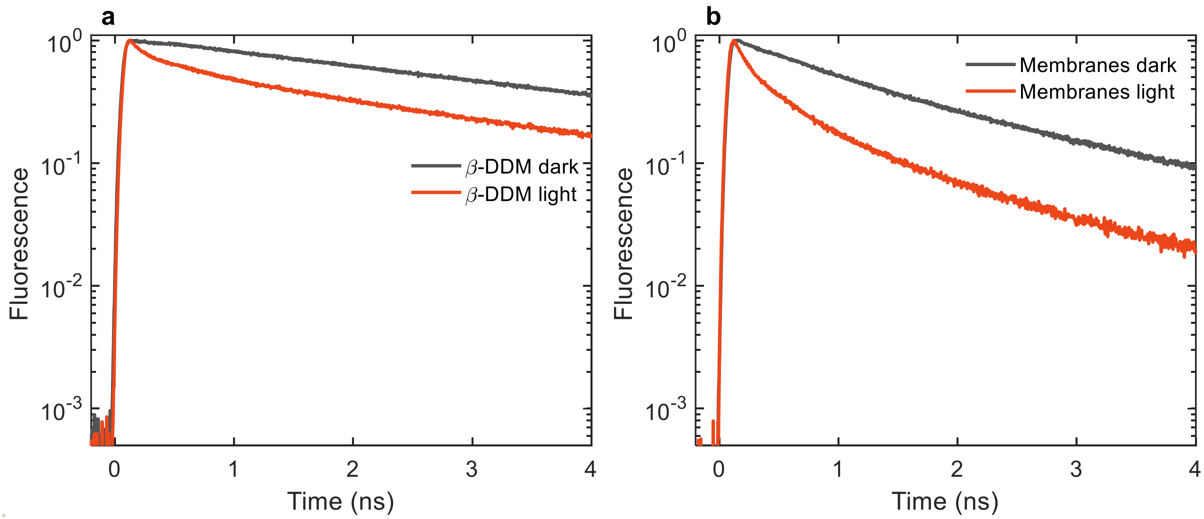

Supplementary Figure 6. Fluorescence decay trace of LHCII in different environments at 680 nm emission wavelength with 632 nm excitation light. The fluorescence decays were recorded before and after 30 min of irradiation. **(a)** β-DDM, **(b)** reconstituted membranes.

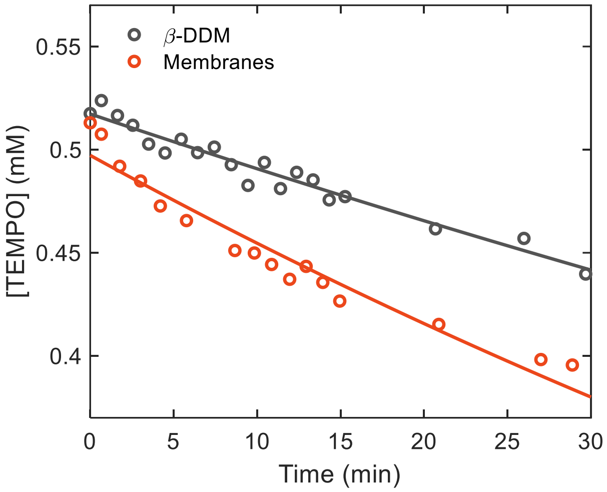

Supplementary Figure 7. Time course of TEMPO concentration calculated from the integrated EPR absorption of LHCII in detergent (β-DDM) and reconstituted membranes containing 0.5 mM TEMPO during 30 min of irradiation.

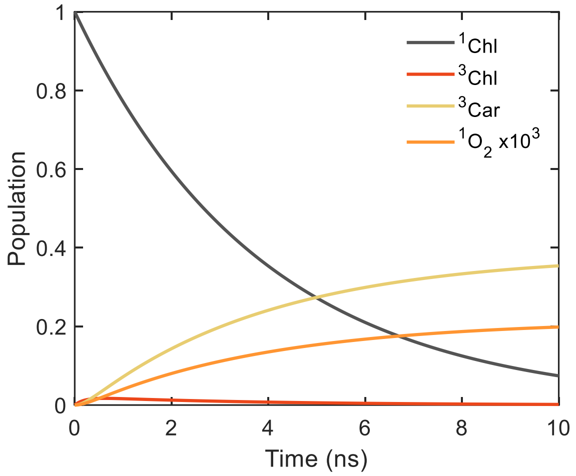

Supplementary Figure 8. Excited-state kinetics of the terminal emitter Chls in LHCII in aerated buffer (rate equation model in the main text). The decay and transfer rate constants (in ns^{-1}) are as follows: $k_D = 0.16$, $k_{ISC} = 0.1$, $k_T = 2\times10^{-6}$, $k_{T-T} = 5$, $k_{Tcar} = 10^{-4}$, $k_{ox} = 2\times10^{9}$ M^{-1} s^{-1} and $[O_2] = 1.4$ mM. The singlet and triplet Chl excited states (black and red curve, respectively) decay with a lifetime of 3.8 ns; Car triplets (gold) and $^1O_2$ (orange) are formed on the same timescale. The quantum yield of $^1O_2$ formation, $\varphi_{ox} = 2\times10^{-4}$.

1.2 Supplementary Tables

Supplementary Table 1. Pigment composition of LHCII 80% acetone extracts from different environments.

LHCII environment	Chl *a* / Chl *b*	Chl *a*+*b* / Car	Car / LHCII
β-DDM	1.31 ± 0.02*	3.5 ± 0.1	4.0 ± 0.1
Reconstituted membranes	1.30 ± 0.02	3.7 ± 0.1	3.8 ± 0.1

* values represent standard error (n = 7–8)

Supplementary Table 2. Photobleaching (PB) rate constants and quantum yields for LHCII in different environments, treated with blue light (incident PFD of 500 µmol photons m^{-2} s^{-1} PAR) from a KL 1500 electronic lamp (Schott, Germany) passed through an SZS–22 glass cutoff filter (580 nm).

LHCII environment	PB after 30 min $\Delta A_{675}/A_{675}$ (%)	PB rate constant k_{pb} (s^{-1})	Quantum yield φ_{pb}
β-DDM	13 ± 3*	(4.3 ± 0.3) × 10^{-5}	(1.7 ± 0.1) × 10^{-5}
Aggregates	26	6.9 × 10^{-5}	2.7 × 10^{-5}
Reconstituted membranes	43 ± 2	(13.5 ± 0.9) × 10^{-5}	(5.2 ± 0.3) × 10^{-5}

* values represent standard error (n = 1–3)
